# A systematic assessment of the current capacity to act in nutrition in West Africa: cross-country similarities and differences

**DOI:** 10.3402/gha.v7.24763

**Published:** 2014-07-16

**Authors:** Roger Sodjinou, William K. Bosu, Nadia Fanou, Lucie Déart, Roland Kupka, Félicité Tchibindat, Shawn Baker

**Affiliations:** 1 UNICEF Regional Office for West and Central Africa, Dakar, Senegal; 2 West Africa Health Organization (WAHO), Bobo-Dioulasso, Burkina Faso; 3 UNICEF Cameroon, Yaoundé, Cameroon; 4 Bill and Melinda Gates Foundation, Seattle, WA, USA

**Keywords:** capacity development, nutrition, undernutrition, nutrition workforce, West Africa

## Abstract

**Background:**

Although it is widely accepted that lack of capacity is one of the barriers to scaling up nutrition in West Africa, there is a paucity of information about what capacities exist and the capacities that need to be developed to accelerate progress toward improved nutrition outcomes in the region.

**Objective:**

To systematically assess the current capacity to act in nutrition in the West Africa region and explore cross-country similarities and differences.

**Design:**

Data were collected from 13 West African countries through interviews with government officials, key development partners, tertiary-level training institutions, and health professional schools. The assessment was based on a conceptual framework of four interdependent levels (tools; skills; staff and infrastructure; and structures, systems and roles). In each of the surveyed countries, we assessed capacity assets and gaps at individual, organizational, and systemic levels.

**Results:**

Important similarities and differences in capacity assets and gaps emerged across all the surveyed countries. There was strong momentum to improve nutrition in nearly all the surveyed countries. Most of the countries had a set of policies on nutrition in place and had set up multisectoral, multi-stakeholder platforms to coordinate nutrition activities, although much remained to be done to improve the effectiveness of these platforms. Many initiatives aimed to reduce undernutrition were ongoing in the region, but there did not seem to be clear coordination between them. Insufficient financial resources to implement nutrition activities were a major problem in all countries. The bulk of financial allocations for nutrition was provided by development partners, even though some countries, such as Niger, Nigeria, and Senegal, had a national budget line for nutrition. Sporadic stock-outs of nutrition supplies were reported in most of the countries as a result of a weak logistic and supply chain system. They also had a critical shortage of skilled nutrition professionals. There was limited supervision of nutrition activities, especially at lower levels. Nigeria and Ghana emerged as the countries with the greatest capacities to support the expansion of a nutrition workforce, although a significant proportion of their trained nutritionists were not employed in the nutrition sector. None of the countries had in place a unified nutrition information system that could guide decision-making processes across the different sectors.

**Conclusions:**

There is an urgent need for a shift toward wider reforms for nutrition capacity development in the West Africa region. Addressing these unmet needs is a critical first step toward improved capacity for action in nutrition in the region.

Undernutrition remains a major public health concern in West Africa, where the prevalence of child stunting is one of the highest in the world and childhood underweight is the leading risk factor for disability and premature deaths (1). Moreover, micronutrient deficiencies – mainly vitamin A, zinc, iodine, and iron – impair childhood development and increase risk of infection and death. The disease burden attributable to undernutrition in West Africa is one of the greatest in the world (2). In addition, a number of countries in the region are also experiencing a rise in obesity and diet-related chronic diseases (3).

There is currently strong momentum to accelerate progress toward improved nutrition outcomes in West Africa. With the exception of Cape Verde, all countries in the West Africa region have now signed up to the Scaling Up Nutrition (SUN) movement. This provides a unique opportunity to improve overall nutrition governance in the region. However, political commitment is likely to be short-lived if it is not translated into tangible actions which address the major impediments to the implementation of nutrition interventions (4). It is well acknowledged that lack of capacity is one of the factors hindering West African countries from making progress in nutrition. There have been recent calls for long-term support and investments in nutrition capacity development (4, 5). In 2009, the Assembly of Health Ministers of the Economic Community of West African States (ECOWAS) also adopted a resolution for action in nutrition, which emphasizes the urgent need to develop capacity for nutrition in West Africa. The resolution also calls on partners to support the West African Health Organization (WAHO) to implement actions to build the needed capacity to accelerate progress for nutrition in the region (6). As a result, the West Africa Nutrition Capacity Development Initiative (WANCDI) was launched in 2013 in an effort to bridge the nutrition capacity gap in the West Africa region.

Although it is widely accepted that there is an urgent need to develop capacities for nutrition in West Africa, there is a paucity of information about what capacities exist and what capacities need to be developed to accelerate progress toward improved nutrition outcomes. Moreover, little has been done to strengthen nutrition capacity at organizational and systemic levels. Previous capacity development efforts have mainly focused on strengthening human capacity for nutrition. Contrary to a common belief, capacity development goes beyond the training of individuals (7). It encompasses various interdependent levels (e.g. individual, organizational, and systems) and it is the synergies among these levels that lead to positive outcomes (7). There is indeed robust evidence that no single action, for example, creating a workforce capacity for nutrition, will lead to sustainable capacity to act in nutrition (4, 8).

The World Health Organization has conducted a landscape analysis of the capacity to scale up nutrition actions in some West African countries (9, 10). However, their analysis focused mainly on five high-burden West African countries (Burkina Faso, Cote-d'Ivoire, Ghana, Mali, and Guinea). A region-wide assessment would be more useful in identifying key priority areas and so guide the development of a regional nutrition capacity development strategy.

This paper presents a systematic assessment of the current capacity of West African countries to act in nutrition. It explores cross-country similarities and differences in capacity assets and gaps.

## Methods

This qualitative study is part of a larger capacity needs assessment conducted in the West Africa region. The assessment was conducted within the framework of the WANCDI implemented under the auspices of WAHO.

The West Africa region was defined according to the United Nations country classifications (11). It has a total 16 countries comprising Mauritania and the 15 member states of the ECOWAS. The region has a total population of about 340 million people and covers an area of approximately 6.1 million square km (12). The region can be categorized into three major language groups:

1. Anglophone countries (Ghana, Liberia, Nigeria, Sierra Leone, and The Gambia)
2. Francophone countries (Benin, Burkina Faso, Cote d'Ivoire, Guinea, Mali, Mauritania, Niger, Senegal, and Togo)
3. Lusophone countries (Cape Verde and Guinea-Bissau).

### Data collection

Data were collected in 13 of the 16 West African countries between March and August 2013. We did not conduct field visits in three countries (Cape Verde, Guinea-Bissau, and Gambia) because our preliminary contacts with in-country key informants revealed that tertiary-level nutrition training activities were either non-existent or were not well-developed. Data were collected by three experienced interviewers through self-administered questionnaires or face-to-face interviews during country visits. Data were completed (either manually or electronically) by the interviewers in the presence of respondents during in-person meetings. Completed self-administered questionnaires were emailed to the research team. Interviews were not audio or videotaped, so data transcription was not performed.

Data for this assessment were derived from five main sources:

1. Interviews with government officials in charge of nutrition: A semi-structured questionnaire (available upon request) was administered to the nutrition focal person in each of the surveyed countries to explore the three interdependent levels (individual, organizational, and systemic) of nutrition capacity development. The questionnaire was structured around four key elements: workforce planning and leadership, management of workforce, work environment, and human resources.
2. Interviews with key stakeholders involved in nutrition: Data were also collected from other sources to support the findings from discussions with government officials. Interviews were conducted in each of the surveyed countries with key stakeholders, namely non-governmental organizations, United Nations (UN) agencies, and donors. A strengths, weaknesses, opportunities, and threats (SWOT) analysis was conducted with all stakeholders.
3. Interviews with nutrition training institutions and health professional schools: We conducted a detailed inventory of the current capacity for nutrition training in each of the surveyed countries. Data were collected using a semi-structured questionnaire that was either administrated during face-to-face discussion or self-administrated by tertiary-level training institutions offering nutrition degree programs. Health professional schools offering nutrition courses as part of their training curricula were also interviewed. Information was collected on the content and status of nutrition courses; profile of faculty members; students and graduates; funding; facilities; prevailing teaching methods; school ownership; institutional affiliation; main funding source; and institutional collaborations. Details about this inventory have been described elsewhere (13).
4. Country presentations from the ECOWAS nutrition meeting: Data were also collected during the ECOWAS nutrition forum mid-term review meeting held in Monrovia, Liberia, 26–27 November 2013. The workshop was organized by WAHO and was attended by the nutrition focal points of all the surveyed countries. Data were also gathered from country presentations made by the nutrition focal points during the meeting.
5. Literature review and internet searches: Besides the interviews, we did a comprehensive literature review that looked beyond individual training needs and focused on previous reports addressing nutrition capacity development in West Africa. We also checked several web portals, including the SUN movement website (14).

### Data analyses

**Fig. 1:**
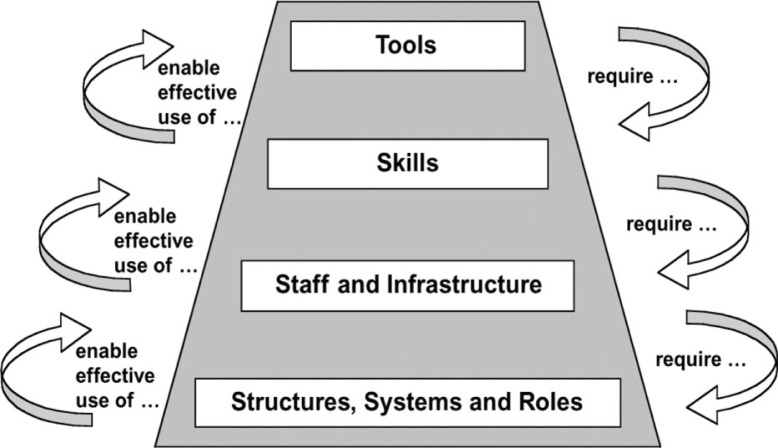
Capacity building pyramid-Potter and Brough (7).

Data were entered into Microsoft Excel 2010. They were analyzed and interpreted using content analysis. We used the analytical framework developed by Potter and Brough (7) to systematically assess the current capacity to act in nutrition at the individual, organizational, and systemic levels (Fig. 1). The framework considers capacity development as a hierarchy of needs, portrayed as a pyramid of four interdependent levels, starting from the ‘easier to achieve’ at the apex of the pyramid (tools, skills, staff, and infrastructure) to the ‘much harder’ to implement at its bottom (systems, structures, and roles). The different levels of the pyramid are further divided into nine interdependent sub-components. The topics covered in each of the surveyed countries, for each sub-component, are highlighted in Table 1.

**Table 1:** Capacity development issues covered in each of the surveyed countries

Level of capacity^a^	Components^a^	Sub-components^a^	Issues covered in each of the surveyed countries
Individual	Tools	Performance capacity	• Are there sufficient nutrition job aids, equipment, and supplies in health facilities? • Is there enough funding for nutrition activities?
	Skills	Personal capacity	• Are nutrition service providers sufficiently skilled to do the job? • Does the training received by nutrition service providers adequately prepare them for nutrition actions?
Organizational	Staff	Workload capacity	• Are there enough nutrition service providers to do the job? • Are roles and responsibilities well-defined in the job descriptions of nutrition service providers? • Is there a formal cadre of placement of nutritionists?
		Supervisory capacity	• Are there clear reporting systems in place? • Are there clear lines of accountability?
	Infrastructure	Facility capacity	• Are there enough health facilities or nutrition service delivery points? • Are health facilities adequately equipped for the provision of nutrition services?
		Support service capacity	• Are there enough training centers to support the development of nutrition workforce? • What is the current capacity for nutrition training?
Systemic	Structure	Structural capacity	• Are there nutrition policies and plans in place? • Are there coordination platforms and mechanisms in place? • What is the internal management structure?
	Systems	Systems capacity	• How is the recruitment of nutrition service providers done? • How nutrition activities are planned at national, regional, and district level?
	Roles	Role capacity	• Is there a sound monitoring and evaluation system in place to support decision-making process?

a Derived from Potter and Brough (7).

### Ethical considerations

Administrative authorization was sought by sending a formal correspondence to the head of all of the targeted institutions prior to data collection. We did not seek ethical approval for the study as there was no data collection on human subjects. All respondents were fully informed about the purposes of the study. They gave their full verbal consent before participating in the study.

## Results

In the 13 surveyed countries, semi-structured interviews were conducted with 44 government officials and 51 stakeholders, namely representatives of non-governmental organizations, UN agencies, and donors (Table 2). We identified 305 nutrition degree programs organized by 113 tertiary-level institutions and 127 training programs organized by 52 health professional schools.

**Table 2:** Data collection methods and participating institutions

	Interview with government officials			Interview with nutrition training institutions		Interview with health professional schools	
				
Countries	Number of interviews conducted	Number of participants	Number of interviews with key stakeholders	Number of participating institutions	Number of training programs assessed	Number of participating institutions	Number of training programs assessed
Benin	4	5	10	2	13	2	2
Burkina Faso	1	3	2	7	26	6	22
Cote-d'Ivoire	1	4	1	4	9	1	2
Ghana	1	2	5	5	15	1	1
Guinea	1	2	1	12	24	6	9
Liberia	3	6	7	2	2	1	1
Mali	2	6	8	13	18	7	12
Mauritania	2	4	3	5	15	4	13
Niger	2	4	2	23	57	8	27
Nigeria	2	2	2	24	68	3	3
Senegal	2	4	8	9	34	9	24
Sierra Leone	1	1	1	5	22	4	11
Togo	1	1	1	2	2	0	0
Total	23	44	51	113	305	52	127

### Capacity at individual level (tools and skills)

We report the findings relating to logistics, information management, funding, and the quality of nutrition training. In all the 13 surveyed countries, nutrition service providers, in general, had the needed equipment (weighing scales, measuring boards, mid-upper arm circumference tapes, behavior change materials, etc.) and supplies to perform nutrition-related tasks, although they were not available everywhere or at all service delivery points. Equipment and nutrition supplies (especially ready-to-use therapeutic foods) were mainly provided by development partners. Concerted efforts had been made to ensure the availability of these nutrition products at all service delivery points. Sporadic stock-outs of nutrition supplies were reported in most of the countries as a result of a weak logistic and supply chain system, poor forecasting of nutrition supplies at lower levels, and inadequate transportation systems. As of the time of our study, development partners were playing a strong role in the procurement and distribution of these nutrition supplies.

A common challenge for all 13 countries was the scarcity of financial resources for nutrition activities (Table 3). Although some countries, such as Ghana, Mauritania, Nigeria, and Senegal, experienced an increasing trend in the budget allocated to nutrition activities over the past years, there still remained some important financial gaps. At the time of our study, only a few countries in the region (Niger, Nigeria, and Senegal) had succeeded in securing a budgetary line for nutrition activities in their national budgets. Other countries, such as Benin, Burkina Faso, Mauritania, and Ghana, were in the process of doing so. As a result, the bulk of resources allocated for nutrition activities in the region was provided by donors and development partners. In Burkina-Faso, some nutrition activities were financed through a pooled fund portfolio, which contained mostly funds from donors and development partners.

Another common observation was the difficulty in getting accurate information about nutrition financing. The fact that resources come from various sources and cut across many sectors made it difficult to track investments and expenditures on nutrition. In Ghana for example, each government unit carrying out nutrition activities had its own budget that was often embedded in a higher level budgetary category. Efforts were underway in most of the countries to improve clarity in overall nutrition budgeting. In Ghana, a sub-committee on resource mobilization had been established, within the Cross-Sectoral Planning Group (CSPG) of the national SUN movement secretariat, to track resource allocation for nutrition in the country.

Inadequate human resources for nutrition were a major barrier to the implementation of nutrition activities (Table 3). In all the 13 countries, there was a critical shortage of skilled nutrition professionals, especially at lower levels. Nutrition graduates did not have practical experience and were not adequately prepared to tackle real-life nutrition issues. There were indeed important gaps in tertiary-level nutrition training in all the 13 countries. Nutrition training curricula were not aligned to regional nutrition priorities. In addition, there did not seem to be a coordinated approach toward harmonization of nutrition training curricula. Existing nutrition degree programs did not provide a full coverage of all essential aspects of human nutrition. Details about the current capacity for nutrition training in the surveyed countries have been reported elsewhere (13).

In the absence of a critical mass of skilled nutrition professionals, the bulk of nutrition work was done by health workers who did not have a good understanding of the application of nutrition principles to public health or clinical practice. As a result, they did not have broad enough skills to provide quality nutrition services. We noted that some countries were taking steps to enhance the performance of health workers through in-service training and mentoring in nutrition. However, these efforts were mainly oriented toward the management of acute malnutrition. In addition, continuing training efforts were mainly concentrated on staff at national and regional levels, leaving staff at lower levels with inadequate capacities in nutrition.

**Table 3:** Similarities and differences in capacity assets and gaps across all the surveyed countries

Country	Nutrition-related policies and plans	Coordination of nutrition activities	Localization of government body that oversees nutrition	Funding for nutrition activities	Adequacy of nutrition workforce	Cadre of placement of nutritionists	Nutrition information systems
Benin	Existence of a Strategic Plan for the Development of Food and Nutrition, with a National Strategy on Infant and Young Child Feeding (IYCF).	Existence of a national multi-sectoral, multi-stakeholder platform of nutrition activities, the *Conseil National de l'Alimentation et de la Nutrition* (CAN). Weak coordination of nutrition activities at lower levels.	At the level of President of Republic.	Inadequate resources for nutrition activities. The government was taking steps to establish a budget line for nutrition.	Limited capacity to produce nutrition graduates. While about 70 nutritionists are needed every year in the country, the current output is just at 50. Critical shortage of skilled nutritionists.	No formal cadre of placement in place.	No single, multi-sectoral information system for nutrition to guide decision-making processes across the different sectors.
Burkina Faso	Existence of a National Policy for Nutrition, with a Strategic Plan for Nutrition for 2010–2015 and a Scaling-Up Plan for IYCF (2014–2025).	The *Conseil National de Concertation en Nutrition* (CNCN) is the multi-sectoral, multi-stakeholder coordination body of nutrition activities. Weak coordination of nutrition activities at lower levels.	Within the Ministry of Health.	Inadequate financial resources for nutrition activities. High dependency on donor resources. The government was taking steps to establish a budget line for nutrition.	Exceptionally weak capacity to produce nutrition graduates. While about 120 nutritionists are needed every year in the country, the current output is just at 15. Critical shortage of nutritionists.	No formal cadre of placement in place.	No single, multi-sectoral information system for nutrition to guide decision-making processes across the different sectors.
Cote-d'Ivoire	No policy document on nutrition in place at the time of data collection.	No multi-sectoral, multi-stakeholder coordination platform in place at the time of data collection. The country was however taking steps to establish a national multi-sectoral, multi-stakeholder platform on nutrition.	Within the Ministry of Health.	Inadequate financial resources for nutrition programs. Insufficient government investments in nutrition. Lack of clarity on funding flows on nutrition.	Exceptionally weak capacity to produce nutrition graduates. While about 165 nutritionists are needed every year in the country, the current output is just at 12. Critical shortage of nutritionists.	No formal cadre of placement in place.	No single, multi-sectoral information system for nutrition to guide decision-making processes across the different sectors.
Ghana	Existence of a National Nutrition Policy (2013–2017), with a set of nutrition plans and guidelines (Imagine Ghana free of malnutrition; Breastfeeding policy; IYCF Strategy; Integrated Anemia Strategy; Vitamin A policy).	Nutrition activities coordinated across sectors through the Cross-Sector Planning Group for Nutrition (CSPG). Coordination mechanisms at lower levels were being strengthened and institutionalized at the time of data collection.	Under the National Development Planning Commission.	Important gaps remained despite increased trend in the budget allocated to nutrition over the past years. The government was taking steps to establish a budget line for nutrition. Lack of clarity on funding flows on nutrition.	Adequate capacity to produce nutrition graduates. In 2013, there were about 275 undergraduate and 50 graduates enrolled in the country. Inadequate distribution of the nutrition workforce and critical shortage of skilled nutritionists at lower levels.	Existence of a formal cadre of placement through the civil service commission.	No single, multi-sectoral information system for nutrition to guide decision-making processes across the different sectors.
Guinea	Existence of a National Policy on Food and Nutrition, with a Strategic Implementation Plan.	No multi-sectoral, multi-stakeholder coordination platform in place at the time of data collection. The Ministry of Health and Public Hygiene continued to coordinate nutrition activities.	Within the Ministry of Health and Public Hygiene.	Scarcity of financial resources for nutrition. High dependency on donor resources. Lack of clarity on funding flows on nutrition.	Exceptionally weak capacity to produce nutrition graduates. While about 80 nutritionists are needed every year in the country, the current output is less than 10.	No formal cadre of placement in place.	No single, multi-sectoral information system for nutrition to guide decision-making processes across the different sectors.
Liberia	No national nutrition policy in place at the time of data collection. Existence of a 5-year Integrated Nutrition Work Plan under the National Health Policy plan (2011–15).	The Ministry of Health continued to coordinate nutrition activities. The National Nutrition Coordination Committee is the coordination body. County level coordination platforms were being established at the time of data collection.	Within the Ministry of Health.	Scarcity of financial resources for nutrition. Donor funding far outweighed government funding. Lack of clarity on funding flows on nutrition. Efforts were underway to improve the situation.	Exceptionally weak capacity to produce nutrition graduates. While about 30 nutritionists are needed every year in the country, no nutrition degree program is in place to support the production of skilled nutrition professionals.	Existence of a formal cadre of placement through the civil service commission.	No single, multi-sectoral information system for nutrition to guide decision-making processes across the different sectors.
Mali	Existence of a National Policy of Nutrition, with a costed multi-sectoral nutrition action plan that was being validated at the time of data collection.	The *Conseil National de la Nutrition* and the *Comité Technique Inter-sectoriel de Nutrition* are the convening bodies for nutrition. No multi-sectoral, multi-stakeholder coordination platform in place at the time of data collection. Efforts were underway to fill this gap.	Within the Ministry of Health.	Inadequate resources for nutrition activities. Nutrition activities were mainly funded by development partners. Lack of clarity on funding flows on nutrition. Efforts were underway to improve clarity in overall nutrition budgeting.	Exceptionally weak capacity to produce nutrition graduates. While about 120 nutritionists are needed every year in the country, no nutrition degree program is in place to support the production of skilled nutrition professionals.	No formal cadre of placement in place.	No single, multi-sectoral information system for nutrition to guide decision-making processes across the different sectors.
Mauritania	Existence of a National Policy for the Development of Nutrition and an Intersectoral Action Plan for Nutrition (PAIN).	The *Comité Technique Permanent* is the multi-sectoral, multi-stakeholder platform on nutrition. Coordination at lower levels were done by Regional Nutrition Development Committees. Much remained to be done to improve these effectiveness of these committees.	Under the National Nutrition Development Council.	Important financial gaps despite increased trend in the budget allocated to nutrition over the past years. The government was taking steps to establish a national budget line for nutrition activities. Lack of clarity on funding flows on nutrition.	Limited capacity to produce nutrition graduates. While about 30 nutritionists are needed every year in the country, the current output is just at 17. Critical shortage of skilled nutrition professionals.	No formal cadre of placement in place.	No single, multi-sectoral information system for nutrition to guide decision-making processes across the different sectors.
Niger	Existence of a National Initiative on Nutrition and Food Security, 3N (*Les Nigériens Nourissent les Nigériens*), a National Policy on Nutrition (2013–2022), and a multi-sectoral nutrition action plan (2013–2017).	The Steering Committee of the Strategic Nutrition Program under the Inter-departmental Orientation Committee of the ‘3N’ initiative, is the multi-stakeholder platform on nutrition.	Within the Prime Minister's office and the Ministry of Health.	Increased trend in the budget allocated (either by the government or donors) to nutrition over the past years. Existence of a national budget line for nutrition activities. Lack of clarity on funding flows on nutrition. Efforts were underway to improve clarity in overall nutrition budgeting.	Limited capacity to produce nutrition graduates. While about 120 nutritionists are needed every year in the country, the current output is at 86. Shortage of skilled nutrition professionals.	Existence of a formal cadre of placement through the civil service commission.	No single, multi-sectoral information system for nutrition to guide decision-making processes across the different sectors.
Nigeria	Existence of a National Food and Nutrition Policy that was being reviewed at the time of data collection, and a Costed National Strategic Framework and Plan of Action for Nutrition.	No multi-sectoral, multi-stakeholder coordination platform in place at the time of data collection. The Ministry of Health continued to coordinate nutrition activities. Efforts were underway to establish an effective coordination mechanism at national level and make functional existing committees on nutrition.	Within the Ministry of Health.	Increased trend in the budget allocated to nutrition over the past years. Existence of a national budget line for nutrition activities. Lack of clarity on funding flows on nutrition. Efforts were underway to improve clarity in overall nutrition budgeting.	Adequate capacity to produce nutrition graduates. In 2013, there were about 1,000 undergraduate and 200 graduates enrolled in the country. Inadequate distribution of the nutrition workforce and critical shortage of skilled nutritionists at lower levels.	Existence of a formal cadre of placement through the civil service commission.	No single, multi-sectoral information system for nutrition to guide decision-making processes across the different sectors.
Senegal	Existence of a National Nutrition Policy (*Lettre de Politique de Nutrition*) that was being reviewed at the time of data collection. Efforts were underway to develop a strategic multi-sectoral nutrition action plan.	The *Cellule de Lutte contre la Malnutrition (CLM)* ensures overall and cross-sector coordination of nutrition activities in the country.	Within the Prime Minister's office.	Increased trend in the budget allocated to nutrition over the past years. Existence of a national budget line for nutrition activities. Lack of clarity on funding flows on nutrition.	Limited capacity to produce nutrition graduates. While about 100 nutritionists are needed every year in the country, the current output is just at 5. Inadequate distribution of the nutrition workforce. Critical shortage of skilled nutritionists.	No formal cadre of placement in place.	No single, multi-sectoral information system for nutrition to guide decision-making processes across the different sectors.
Sierra Leone	Existence of a Food and Nutrition Policy that was in draft form at the time of data collection. Existence of a costed multi-sectoral Food and Nutrition Policy Implementation Plan (2013–2017).	The convening bodies for nutrition are the National Food and Nutrition Security Steering committee (a high-level government body) and the National Food and Nutrition Security Technical Committee (a technical convening body).	Within the Vice President's office and the Ministry of Health.	Inadequate financial resources for nutrition activities. Nutrition activities were mainly funded by donors. Lack of clarity on funding flows on nutrition. Efforts were underway to improve clarity in overall nutrition budgeting.	Good capacity to produce nutrition graduates. While about 40 nutritionists are needed every year in the country, the current output is at 45. Inadequate distribution of the nutrition workforce and critical shortage of skilled nutritionists at lower levels.	Existence of a formal cadre of placement through the civil service commission.	No single, multi-sectoral information system for nutrition to guide decision-making processes across the different sectors.
Togo	Existence of a National Policy on Food and Nutrition, with a National Strategic Plan for Food and Nutrition (2012–2016).	No multi-sectoral, multi-stakeholder coordination platform in place at the time of data collection. The Ministry of Health continued to coordinate nutrition activities.	Within the Ministry of Health.	Scarcity of financial resources for nutrition activities. Donor funding far outweighed government funding. Lack of clarity on funding flows on nutrition.	Exceptionally weak capacity to produce nutrition graduates. About 50 nutritionists are needed every, but no nutrition degree program is in place in the country. Critical shortage of skilled nutrition professionals.	No formal cadre of placement in place.	No single, multi-sectoral information system for nutrition to guide decision-making processes across the different sectors.

### Capacity at organizational level (staff and infrastructure)

The results on capacity at the organizational level relate to the production of human resources, staff placement, and supervision. A common characteristic for all the surveyed countries was a critical shortage of nutritionists (Table 3). Although at least 2,000 nutritionists are needed every year in the region, the current output was just at 700. The surveyed countries differed, however, in their capacity to support the expansion of nutrition workforce. Although the capacity to produce nutrition graduates was weak in most of the countries, there seemed to be a reasonable number of trained nutritionists in Nigeria, Ghana, and Sierra Leone. In 2013, there were about 1,000 undergraduate and 200 graduates enrolled in Nigeria, whereas Ghana had about 275 undergraduates and 50 graduates enrolled in nutrition. In Sierra Leone, the current output (45) outweighed the annual need of the country. However, the distribution of the nutrition workforce was inadequate in these three countries. In Nigeria, for example, the vast majority of trained nutritionists worked in urban areas. In addition, a significant proportion of them worked outside the nutrition sector. In Ghana, there was a critical shortage of nutritionists at the district level. This situation put considerable demand on health workers who had to perform nutrition-related tasks in the absence of skilled nutrition professionals. Because these health workers were overwhelmed by other equally important public health tasks, they tended to pay insufficient attention to nutrition. As a result, there was a lack of commitment to nutrition activities in all the surveyed countries.

In all the surveyed countries, training institutions supporting the development of nutrition workforce faced many challenges with teaching resources. They were poorly staffed, financially constrained, and had weak research capacity. They lacked teaching materials and equipment, infrastructure, libraries, and access to advanced technology resources. In most of the countries, there did not seem to be a coordinated effort toward interactions between academia, policy makers, and program managers. As a result, nutrition training institutions operated in silos and rarely participated in policy development or service delivery.

The countries also differed significantly in their approach with regard to staff placement (Table 3). Although some countries, such as Ghana, Liberia, Niger, Nigeria, and Sierra Leone, had a formal cadre of placement of nutritionists through the civil service commission, the other surveyed countries did not yet have a formal cadre of recognized nutritionists. A common pattern for all 13 countries was the lack of regulation of the nutrition profession. None of the countries had a set of minimum standards to qualify as a nutritionist in place. As a result, professionals from other sectors or people with little background in nutrition could be recruited and given the title of nutritionist.

Most of the countries faced challenges in supervising nutrition activities at lower levels. We noted that supportive supervision and mentoring on the job were not systematically or widely carried out. There was limited monitoring because of inadequate logistics and funding. All countries agreed that the provision of additional means of transport would enhance supervision and thereby strengthen program implementation.

### Capacity at systemic level (structures, systems, and roles)

The results on capacity at systemic level relate to governance, coordination mechanisms, and information management. There was strong momentum to improve nutrition in most of the countries. This was illustrated by the existence of a set of policies and plans on nutrition, which are used to prioritize actions and track progress toward the achievement of stated objectives (Table 3). However, much remained to be done to align the efforts of all stakeholders to these national nutrition policies and plans. The fact that all the surveyed countries had signed up to the SUN movement was a positive move toward improved nutrition governance in the West Africa region.

Most of the countries had also set up national multisectoral, multi-stakeholder platforms that brought together all the major stakeholders in the nutrition area. These platforms offered the opportunity to share lessons and best practices, as well as to reduce duplication of efforts. Their location differed in the surveyed countries. In Benin for example, the coordinating body was placed directly under the Office of the President, whereas it was within the Prime Minister's office in Niger and Senegal. Ghana and Mauritania had set up a separate government body that oversaw nutrition activities at national level. The Ministry of Health continued to coordinate nutrition activities in Burkina Faso, Cote d'Ivoire, Liberia, Mali, and Nigeria. Cote d'Ivoire and Liberia were, however, taking steps to establish, within the framework of their involvement in the SUN movement, a multisectoral, multi-stakeholder coordination mechanism for nutrition activities. Other new initiatives aimed at improving nutrition security were also ongoing in the surveyed countries. The Renewed Efforts Against Child Hunger and undernutrition (REACH) had also gained momentum in five countries in West Africa (Ghana, Mali, Mauritania, Niger, and Sierra Leone). The European Union and other development partners had also launched the Global Alliance for Resilience Initiative (AGIR).

A common weakness in all countries was the lack of or inadequate coordination mechanisms at lower levels. The cross-disciplinary nature of nutrition made it difficult to coordinate nutrition activities, leading to a high degree of fragmentation of nutrition activities across several ministries, departments, and agencies. In Ghana, coordination at the national, regional, and district levels was being strengthened, institutionalized, and empowered to enhance their effectiveness. Countries such as Benin, Burkina Faso, and Nigeria were taking steps to strengthen the coordination of nutrition activities at their policy and operational levels.

The vast majority of the surveyed countries had made considerable efforts to strengthen the nutrition data collection system. Nutrition surveys, using the SMART methodology, were now being conducted on a regular basis in most of the countries and the survey results were being used for decision-making. Nutrition data were also generated through Demographic and Health Surveys (DHS) or Multiple Indicator Cluster Surveys (MICS). Despite these advancements, there were still some unmet needs. None of the countries had as yet succeeded in putting in place a single, multisectoral information system for nutrition that will guide decision-making processes across the different sectors (Table 3). In addition, much remained to be done to improve the accuracy of routine nutrition data collected at lower levels.

## Discussion

In our region-wide assessment of the current capacity to act in nutrition in West Africa, we analyzed key capacity assets and gaps as well as cross-country similarities and differences. Our findings reveal that important efforts have been made over the past years to raise the profile of nutrition to a high priority in the surveyed countries. The nutrition landscape has been evolving in most of the countries in West Africa since 2011. In addition, advocacy efforts are underway to sustain this momentum and keep governments involved. This is a positive move toward the achievement of nutrition-related MDGs in the region. Donors and development partners have also played an important role in this process. The different nutrition initiatives supported by development partners (SUN, REACH, AGIR, etc.) have greatly contributed to the advancement of nutrition in the region.

Despite these positive developments, there are still some challenges and unmet needs. There is a need for clear divisions of tasks between ongoing nutrition initiatives. For example, five of the 13 surveyed countries (Ghana, Mali, Mauritania, Niger, and Sierra Leone) are both SUN and REACH countries. It is critical to ensure good coordination between these two important initiatives as both aim to support country-led processes to improve nutrition governance and enable multisectoral, multi-stakeholders approach to addressing undernutrition (15, 16). This could help prevent potential misunderstandings that may arise. The confusion in multiple nutrition initiatives has been reported in Niger (17).

Our findings also indicate that lack of funding is a major barrier for the implementation of nutrition activities. There is indeed a clear disconnect between national nutrition goals and the resources available for the implementation of nutrition activities. This situation slows the translation of governments’ commitments for nutrition into tangible actions on the ground. We found a high dependency on donor resources for nutrition activities as a result of low government investments. It is critical that governments in the West Africa region take on greater responsibility for nutrition financing.

Another major challenge that was common was the inability to track investments and expenditures on nutrition activities. The importance of having accurate information on nutrition financing cannot be overemphasized. These data are not only crucial for planning purposes (to make projections for nutrition financing), but are also vital in identifying financial gaps and ensuring efficiency and accountability. There is an urgent need to improve the systems for tracking allocations and expenditures for nutrition activities in the surveyed countries. The workshop on costing and tracking investments in nutrition, co-organized by UNICEF and the SUN movement secretariat in Nairobi, Kenya, in November 2013 (18), was a preliminary step in moving toward that direction. Much remains to be done to improve transparency and clarity over nutrition budgeting in the West Africa region. It is gratifying that the SUN movement secretariat continues to work with countries to ensure that nutrition programs are costed and that resources allocated to nutrition activities are tracked (14).

The results of our study indicate that shortage of skilled human resources to deliver nutrition interventions is a major problem in all countries. There is a clear need to support the expansion of the nutrition workforce in the region and provide incentives for nutrition service providers to stay in service. Training institutions should be empowered to support the expansion of the nutrition workforce. In addition, attention should be paid to the pre-service and in-service training in nutrition of health professionals. Our findings also indicate that there is a lack of regulation of the nutrition profession. The need to set a minimum standard to qualify as a nutritionist in the region has been previously stressed (19, 20). It would also be important to create a proper cadre of placement for nutrition service providers and clearly specify their roles and responsibilities.

Overall, our data are consistent with findings in previous studies in some of the West African countries (9, 10). They underscore the urgent need to strengthen capacity for nutrition at individual, organizational, and systemic levels. Our results are also in line with findings in previous studies in other sub-Saharan African countries such as Malawi (21), Namibia (22), and Angola (23). Some common elements are the limited financial resources for nutrition activities, the lack of human resources for nutrition, the lack of pre-service nutrition training programs, the limited capacity to support the expansion of a nutrition workforce, and the lack of cross-sectoral coordination. Clearly, support is urgently needed to build the needed capacity to accelerate progress for nutrition in Africa.

It is not our intention in this paper to make specific recommendations on how to develop capacity for nutrition in the West Africa region. We believe that this should be done in a participatory manner, with the involvement of all stakeholders. For instance, we are planning to convene a regional workshop to discuss the findings from this study and build consensus on the way forward. The ultimate goal of this workshop would be to create a unified discourse between stakeholders on how to strengthen the capacity to act in nutrition in the West Africa region. This will ultimately lead to the development of a consensual regional capacity development strategy that will help shape future nutrition actions.

Our study has several limitations. It was difficult to obtain quantitative data on financial investments in nutrition as they were not readily available in the surveyed countries. In addition, we did not have the opportunity to assess the adequacy of infrastructure for nutrition services through direct observations. Given the rapidly evolving nutrition environment in West Africa, it is possible that some of the information reported in this paper may not fully reflect the current situation on the ground. However, we believe that our study provides a comprehensive view on the current capacity to act in nutrition in the West Africa region.

## Conclusions

Nutrition programs in West Africa are characterized by a critical shortage of skilled human resources, limited funding, high dependency on donor resources, weak logistic and infrastructure systems, and lack of supervision as well as coordination of nutrition activities at lower levels. These factors hamper the ability to act in nutrition in the West Africa region. Addressing these unmet needs is critical in moving toward improved nutrition outcomes in the region. There is an urgent need for a shift toward wider reforms for nutrition capacity development in the West Africa region.
